# The role of Interleukin-22 in severe acute pancreatitis

**DOI:** 10.1186/s10020-024-00826-7

**Published:** 2024-05-15

**Authors:** Hongli Yang, Ruofan Cao, Feifei Zhou, Ben Wang, Qianqian Xu, Rui Li, ChunHua Zhang, Hongwei Xu

**Affiliations:** 1 Department of Gastroenterology, Shandong Provincial Hospital Affiliated to Shandong First Medical University, Ji’nan, Shandong 250021 P.R. China; 2 Medical Science and Technology Innovation Center, Shandong First Medical University, Shandong Academy of Medical Sciences, Jinan, Shandong 250021 P.R. China; 3 Department of Gastroenterology, Cheeloo College of Medicine, Shandong Provincial Hospital, Shandong University, Ji’nan, Shandong 250021 P.R. China; 4 Shandong First Medical University, Ji’nan, Shandong 250117 P.R. China

**Keywords:** IL -22, Sever acute pancreatitis, Inflammation, Autophagy, SAP-induced multiorgan injury

## Abstract

Severe acute pancreatitis (SAP) begins with premature activation of enzymes, promoted by the immune system, triggering a potential systemic inflammatory response that leads to organ failure with increased mortality and a bleak prognosis. Interleukin-22 (IL-22) is a cytokine that may have a significant role in SAP. IL-22, a member of the IL-10 cytokine family, has garnered growing interest owing to its potential tissue-protective properties. Recently, emerging research has revealed its specific effects on pancreatic diseases, particularly SAP. This paper provides a review of the latest knowledge on the role of IL-22 and its viability as a therapeutic target in SAP.

**Supplementary Information:**

The online version contains supplementary material available at 10.1186/s10020-024-00826-7.

## Highlights

1. Interleukin-22 (IL-22) is a member of the IL-10 family of cytokines.

2. IL-22 is a potential indicator for monitoring organ dysfunction and severity of severe acute pancreatitis (SAP).

3. IL-22 acts as a protective factor against acinar cell damage by suppressing autophagy and reducing the infiltration of inflammatory cells.

4. IL-22 alleviate SAP-induced multiorgan injury.

5. IL-22 protect SAP-induced intestinal barrier damage.

**Supplementary Information:**

The online version contains supplementary material available at 10.1186/s10020-024-00826-7.

## Introduction

Incidences of acute pancreatitis (AP), a common inflammatory disease characterized by the destruction of acinar cells that often requires hospitalization (Boxhoorn et al. 2020; Xiao et al. 2016), have increased over the past 10 years. Pancreatitis was the most common diagnosis of gastrointestinal disease in patients treated in the United States(Lankisch et al. 2015). Symptoms of AP are characterized by epigastric pain, nausea, and pain radiating to the back. Most patients experience mild acute pancreatitis (MAP) that resolves spontaneously within one week. However, approximately 20% of patients develop moderate severe acute pancreatitis (MSAP) or severe acute pancreatitis (SAP) with pancreatic or peripancreatic tissue necrosis, systemic inflammatory response syndrome, and organ failure, resulting in a considerable mortality rate of 20–40% (Schepers et al. 2019; Santvoort et al. 2011; Gurusamy et al. 2016; Bang et al. 2020; Banks et al. 2013). There are two peaks in mortality of SAP, the first occurs earlier and is associated with high mortality within two weeks of onset, which is associated with persistent (> 48 h) early organ failure, while the second peak is attributed to sepsis caused by infected necrosis (Johnson 2004). Patients with multiple organ failure stay long in intensive care units, and there require invasive interventions to address local and systemic complications (Working Group IAPAPAAPG 2013). However, no effective therapeutic agent currently exists to mitigate the risks and consequences of SAP (Habtezion et al. 2019; Trikudanathan et al. 2019).

From an immunological perspective, SAP leads to excessive activation of leukocytes and heightened neutrophil infiltration into inflamed tissues, resulting in the release of various inflammatory factors, including interleukins, transforming growth factors, procalcitonin, and tumor necrosis factors, among others (Mofidi et al. 2009; Staubli et al. 2015). Studies have identified immune system activation as a crucial trigger and regulator of inflammatory injury in the pancreas (Sendler et al. 2013; Jiang et al. 2020). As a member of the IL-10 family, which includes IL-10, IL-19, IL-20, IL-24, IL-26, IL-28a, IL-28b, and IL-29, IL-22 has garnered increasing attention due to its potential tissue-protective effects (Ito et al. 2017; Hsueh et al. 2016; Zenewicz et al. 2008; Wolk et al. 2011). IL-22 serves as a crucial signaling molecule involved in various essential physiological processes, including innate immune responses to the inflammatory process. It directly stimulates endothelial cell proliferation, angiogenesis, and migration. IL-22 can also lead to chronic inflammatory diseases, compromised wound healing, and increased susceptibility to infections (Ito et al. 2017; Hsueh et al. 2016; Zenewicz et al. 2008; Wolk et al. 2011). The inflammatory response and cytokine production play a pivotal role in the progression of SAP. Recently, an increasing number of studies have investigated the effects of IL-22 on pancreatic diseases.

In this review, we searched PubMed, Embase, and Cochrane databases for published English language studies of IL-22 and used search terms related to IL-22, acute pancreatitis inflammation, autophagy and SAP-induced multiorgan injury and from the literature retrieved, we summarize recent reports on IL-22 to understand its role in SAP. Our review offers guidance for further exploration of the roles of IL-22 and proposes broadening the treatment of SAP by targeting IL-22.

## IL-22 family and receptors

IL-22, a member of the IL-10 cytokine family, was first discovered in 2000 from a mouse T-cell line stimulated by IL-9-stimulated T-cell (Eyerich et al. 2017). The human IL-22 gene is located on chromosome 12q15 and consists of six alpha helices, comprising a total of 146 amino acids (Wolk and Sabat 2006), and situated near the genes encoding IFN-g and IL-26 (Dumoutier et al. 2000a, b). The IL-22 gene encodes a protein consisting of 179 amino acids (Laure Dumoutier 2000). Prior to secretion of the 146-amino acid cytokine protein, a predicted 33-amino acid signal peptide in the human IL-22 gene sequence is cleaved (Xie et al. 2000). Notably, there is a high degree of homology, approximately 79%, between the human and mouse IL-22 protein sequences (Laure Dumoutier 2000). Initially, IL-22 was found to be constitutively expressed only in the thymus and brain (Dumoutier et al. 2000a, b). However, subsequent studies have revealed its induced expression in various tissues, including the gut, liver, lung, skin, spleen, and pancreas (Sabat et al. 2013). IL-22 is primarily secreted by T cells, including T helper1 (Th1) cells, Th17 cells, Th22 cells, CD8 + T cells, γδ T cells, natural killer T (NKT) cells, CD117 + neutrophils, and group 3 innate lymphoid cells (ILC3s) (Dudakov et al. 2015; Mizoguchi et al. 2017; Cui 2019; Zheng et al. 2006; Duhen et al. 2009; Witte et al. 2010; Zhou et al. 2018). In general, CD4 + T cells and ILC3s are thought to be the main producers of IL-22 in humans, and other T cells, such as CD8 + T cells, γδT cells, and NKT cells, are capable of producing IL-22 when activated, especially in the presence of IL-23 (Ouyang and O’Garra 2019).

**Fig. 1:**
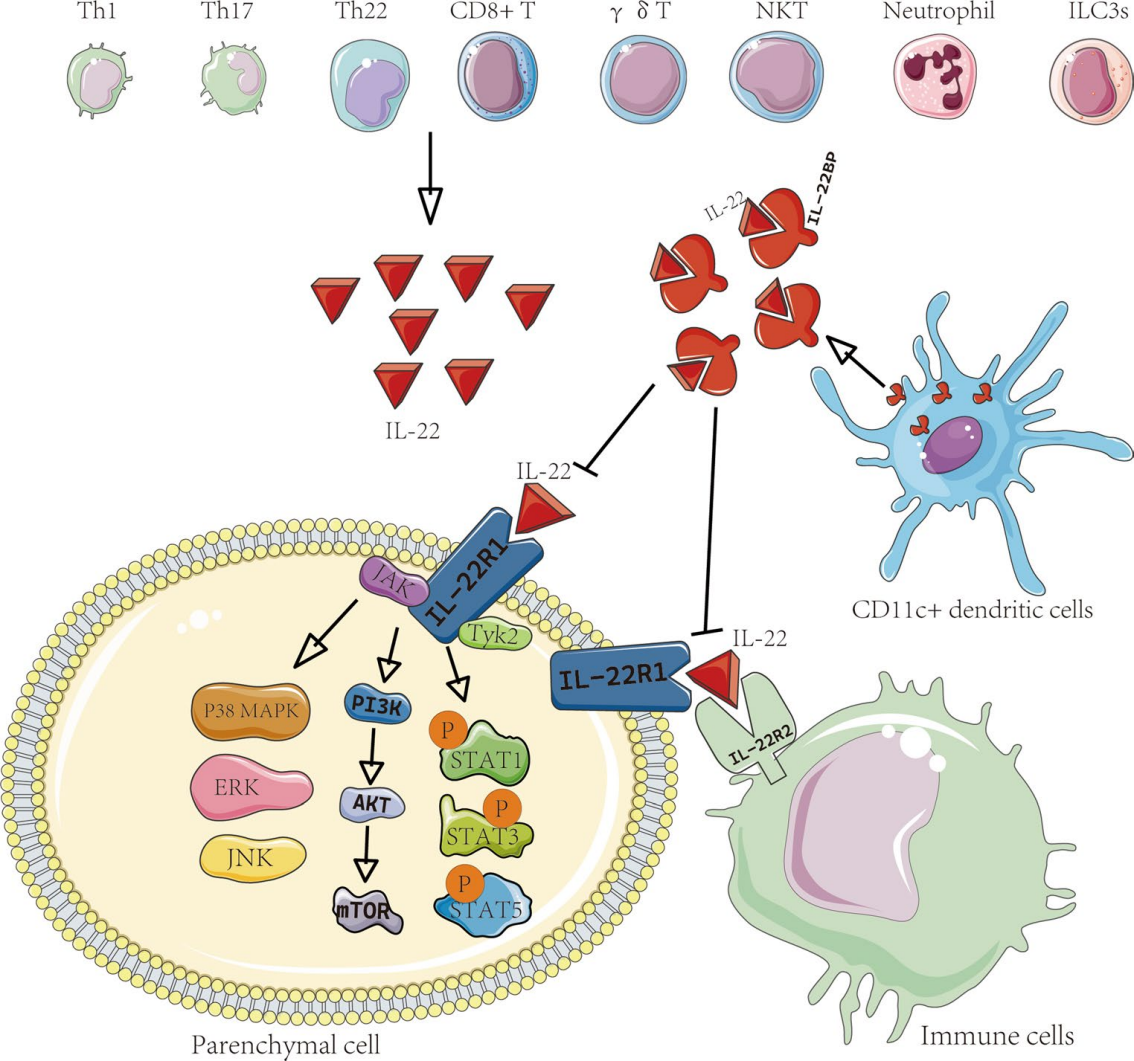
IL-22 family and receptors. IL-22 is predominantly released by Th1 cells, Th17 cells, Th22 cells, CD8 + T cells, γδ T cells, NKT cells, neutrophils, and ILC3s. IL-22 receptor is comprised of two subunits, IL-22R1 and IL-10R2. IL-22R1 is primarily distributed in various tissues, while IL-10R2 is predominantly expressed in immune cells. IL-22 functions by initiating the Jak1, which interacts with IL-22R1 and Tyk2. IL-22 mainly affects STAT signaling pathways (STAT1, STAT3, and STAT5), PI3K-AKT-mTOR and MAPK signaling pathways (p38, ERK, and JNK). IL-22BP is mainly produced by CD11c + cells and binds to IL-22, thereby preventing its binding to the IL-22 receptor

The IL-22 receptor is a heterodimeric receptor complex composed of IL-22R1 and IL-10R2, belonging to the type II cytokine receptor family (Wolk and Sabat 2006). IL-10R2 is predominantly expressed in immune cells, while IL-22R1 is primarily found in various tissues, including the pancreas, keratin-forming cells, respiratory epithelial cells, non-hematopoietic epithelial cells, and fibroblasts in tissues like the kidney, liver, and colon (Dudakov et al. 2015; Wolk et al. 2010). While IL-10R2 has minimal affinity for IL-22, it exhibits a moderate affinity for the IL-22/IL-22R1 complex. Additionally, Jones et al. reported a high affinity between IL-22 and IL-22R1 (Xu et al. 2005). IL-22R1 signals utilize a membrane-bound mechanism to be transmitted from the immune system to tissues (Dudakov et al. 2015; Sabihi et al. 2020). Initially, IL-22 binds to IL-22R1 in a 1:1 ratio, and subsequently, the IL-22/IL-22R1 complex associates with IL-10R2 to initiate downstream signaling. This interaction activates the phosphorylation of Janus kinase 1 (Jak1), which interacts with IL-22R1 and tyrosine kinase 2 (Tyk2). The latter also interacts with IL-10R2. Consequently, the primary signaling targets of IL-22, namely STAT1, STAT3, and STAT5, are activated (Wolk et al. 2010). Furthermore, IL-22 exhibits the capability to activate three major MAPK pathways, namely p38, ERK, and JNK, and some investigations also propose that IL-22, derived from lymphocytes, may specifically impact tissue cells (Mühl et al. 2013). IL-22 binding protein(IL-22BP), identified as a soluble receptor homolog of IL-22 receptor, emerged shortly after the discovery of the role of the IL-22 receptor in the IL-22 receptor complex (Kotenko et al. 2001; Xu 1 et al. 2001), which is a 24-kilodalton protein with an L-shaped structure featuring two consecutive fibronectin-III domains (Zenewicz 2021). The gene responsible for IL-22BP, IL-22R2, is located on chromosome 6 in humans and chromosome 10 in mice (Dumoutier et al. 2001). Evidence indicates that within the gastrointestinal tract, a distinct subset of CD11c + dendritic cells produces IL-22BP (Guendel et al. 2020). (Fig. 1) Consequently, under normal conditions, the low levels of IL-22 produced by immune cells in the gastrointestinal system remain biologically inactive (Guendel et al. 2020; Martin et al. 2014). In Dendritic cells, retinoic acid induces IL-22R2, leading to the upregulation of IL-22BP secretion (Moura et al. 2009; Voglis et al. 2018). Experimental murine models of skin inflammation revealed that during inflammation, the expression of the IL-22R2 gene is downregulated, further suppressing the secretion of IL-22BP (Voglis et al. 2018). IL-22BP functions by binding to IL-22, hindering its interaction with the cellular receptor and displaying inhibitory effects (Lejeune et al. 2002). IL-22BP can inhibit inflammation by binding to IL-22 with significantly higher affinity compared to its receptor IL-22R1 (Dumoutier et al. 2001). These findings imply a significant decrease in IL-22BP levels during inflammation, elucidating the reason for low IL-22 levels in healthy tissues and their rapid elevation during inflammatory responses (Nagalakshmi et al. 2004).

IL-22 regulates innate immune defense, influences cell differentiation, and stimulates the production of antimicrobial peptides and specific chemokines in various tissues (Mizoguchi et al. 2017). It has shown a wide range of biological activities, including tissue regeneration, defense against microbial agents, and regulation of mucosal immunity (Zenewicz and Flavell 2011; Perusina Lanfranca et al. 2020; Manohar et al. 2021). IL-22 mainly plays anti-inflammatory roles in many diseases (Dudakov et al. 2015). A virus-fighting drug candidate, Abx484, dampens intestinal inflammation by stimulating the production of IL-22 in activated macrophages (Chebli et al. 2017). Inflammation and fibrosis in renal tubular cells are also attenuated by IL-22 via the Notch1 signaling pathway (Tang et al. 2020). Furthermore, IL-22 inhibits asthma models induced by house dust mites by inducing Reg3γ (Ito et al. 2017), and suppress allergic inflammation in the lungs, preventing secondary bacterial infections (jia et al. 2021) (Hebert et al. 2020). IL-22 can also alleviate the progression of SAP, which is characterized by local and systemic inflammation. Meng et al. confirmed that IL-22 can mitigate SAP progression and protect the intestinal mucosal barrier (Jin et al. 2022a, b). Therefore, ongoing research has enhanced our understanding of IL-22 and introduced innovative approaches for managing SAP.

## The biological function of IL-22 in SAP

### IL-22 as a novel biomarker of SAP

It should be noted that around one-third of AP patients develop local or systemic complications such as pancreatic hemorrhage, necrosis, secondary infection, peritonitis, and even shock, leading to SAP (Sternby et al. 2019). Because of the swift initiation and advancement of the condition, SAP is linked to a comparatively elevated mortality rate and an overall unfavorable prognosis. Therefore, timely identification of patients at risk of developing SAP is of utmost importance. Numerous models utilizing clinical, biological, and radiological parameters have been devised to predict AP progression. Among these models, C-reactive protein has emerged as one of the most dependable markers within the initial 48 h post-admission period. Nonetheless, its prognostic value upon admission is relatively limited (Working Group IAPAPAAPG 2013). To date, several studies have confirmed the association between IL-22 and SAP, suggesting that IL-22, as a potential prognostic indicator of SAP, is significantly correlated with the degree of organ dysfunction and disease severity (Jin et al. 2022a, b). In a previous study, during the occurrence of AP, the levels of IL-22 in the serum exhibited a substantial increase in comparison to the control group (Czaja 2011). Additionally, the concentration of IL-22 in the serum showed early elevation in patients with AP, and the serum concentration of IL-22 was positively correlated with the severity of AP. Therefore, we can speculate that IL-22 is a valuable prognostic marker for assessing disease progression in patients with AP. A study conducted by Jin et al. (Jin et al. 2022a, b) examined the expression of IL-22 in the peripheral blood of 78 patients with AP. The patient cohort included 11 individuals with mild AP, 32 with moderate AP, and 35 with severe AP. Blood samples were collected within 24 h of admission. It was found that the levels of IL-22 in the serum of patients were significantly higher compared to those in healthy individuals. Furthermore, a notable increase in IL-22 expression was observed in patients with SAP compared to those with MSAP. In addition, patients with AP and gastrointestinal failure exhibited higher serum IL-22 levels than those without gastrointestinal failure. Recent clinical evidence of animal models also confirmed that IL-22 levels increased in the early phase of SAP but declined at a faster rate than the levels of proinflammatory cytokines like IL-6 and TNF-α (Jin et al. 2022a, b). However, the findings by Xue et al. (Xue et al. 2012) revealed a decrease in the expression levels of IL-22 in both cerulean-induced and choline-deficient ethionine-supplemented diet-induced pancreatitis mouse. This reduction in the IL-22 concentration was linked to a significant decrease in the number of IL-22-producing leukocytes, specifically CD4 + T cells. In addition, some studies have found that IL-22 is highly expressed in certain macrophage subsets at the early stage of SAP (Manohar et al. 2021). Given its notable predictive accuracy and swift accessibility, IL-22 has emerged as a promising biomarker among individual indicators, thereby augmenting the predictive efficacy of SAP.

### IL-22 alleviates pancreatic acinar cells damage

IL-22, an anti-inflammatory factor, exerts protective effects against SAP. SAP is characterized as an inflammatory condition affecting the pancreas that involves inflammation, edema, and necrosis of the pancreatic tissues. Among these, necrosis is the primary mechanism of cell death (Boxhoorn et al. 2020; Sung et al. 2009). The pathology of various pancreatic diseases is significantly influenced by the damage inflicted on pancreatic acinar cells. A cascade of events is triggered upon pancreatic acinar cell injury, leading to the release of numerous inflammatory mediators, including cytokines and chemokines. This release, in turn, initiates the recruitment and activation of both innate immune cells (such as neutrophils, macrophages, dendritic cells, mast cells, and NK cells) and adaptive immune cells (such as T cells and B cells) (Peng et al. 2021; Al Mofleh 2008). This initiates an uncontrolled inflammatory response within the acinar cells, eventually leading to the development of systemic inflammatory response syndrome (SIRS), which significantly contributes to the high mortality and unfavorable prognosis observed in pancreatic diseases (Tokoro et al. 2020; Li et al. 2021). Given the well-documented high expression of the IL-22 receptor subunit IL-22R1 in pancreatic acinar cells (UDEEPTA AGGARWAL M-HX, MIKO 2001), it has been suggested that IL-22 potentially serves as a protective factor against acinar cell damage by inhibiting autophagy and inhibiting the infiltration of inflammatory cells.

**Fig. 2:**
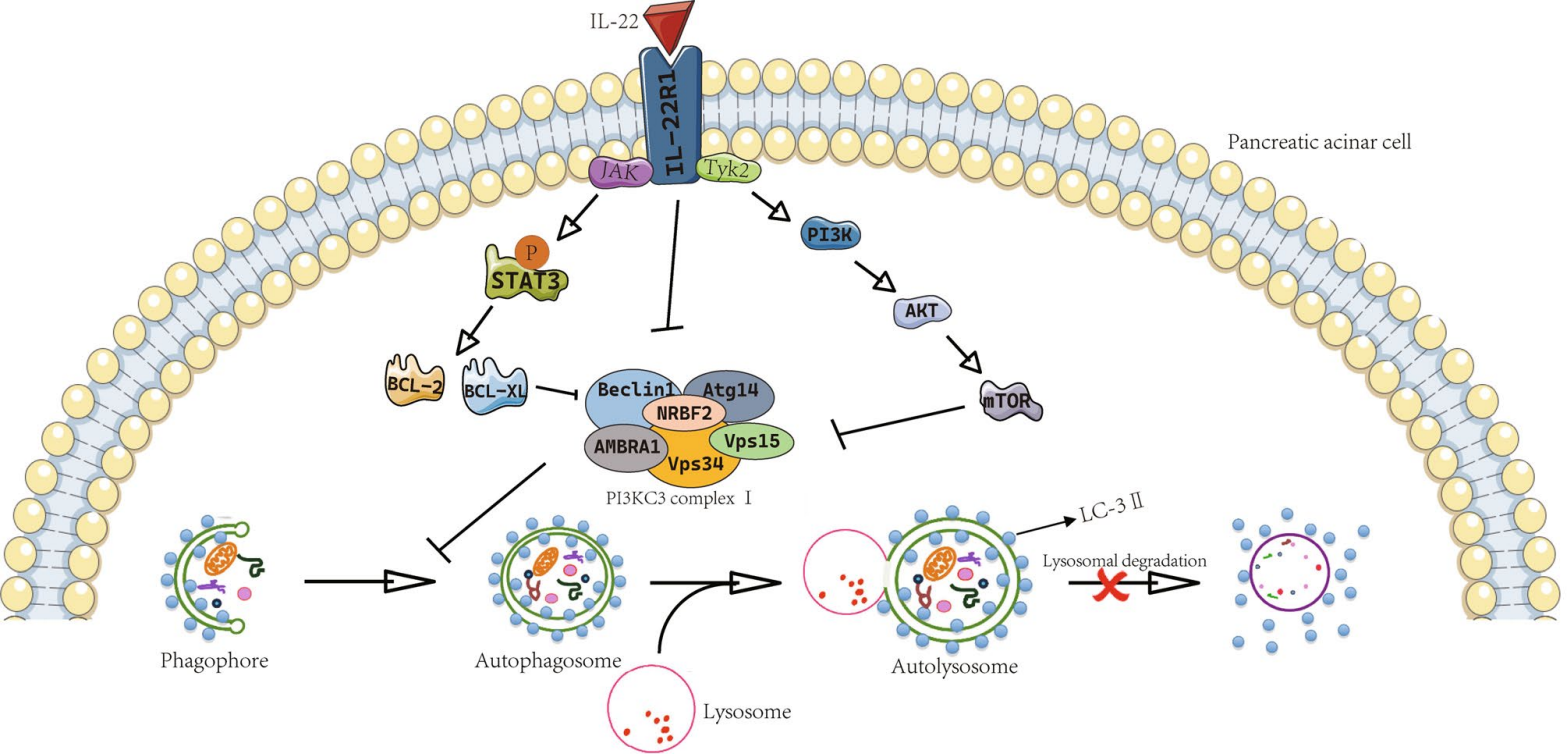
IL-22 alleviates pancreatic acinar cells damage. In pancreatitis, the autophagic flux of pancreatic acinar cells is impaired, mainly due to the dysfunction of autolysosome degradation. IL-22 binds to IL-22R1 in acinar cells to activate PI3K-AKT-mTOR and STAT signaling pathways. mTOR and P-STAT3 up-regulate anti-apoptotic protein, Bcl-2 and Bcl-xL, and inhibit Beclin1 (a member of PI3KC3 complex I) to further inhibit the autophagosome formation

Accumulating evidence indicates that autophagy within acinar cells plays a crucial role in the development of pancreatitis and that IL-22 can inhibit this process. Autophagy involves two sequential processes: first, the formation of autophagosomes that encapsulate organelles targeted for degradation, and second, the fusion of autophagosomes with lysosomes to create autolysosomes, where materials are broken down by hydrolases, including the cathepsin family of proteases. Three primary pathways govern the regulation of autophagy: the inhibitory pathway involving mTOR, the stimulatory pathway involving beclin-1 (a member of PI3KC3 complex I), and the stimulatory pathway involving LC3-II/Atg5-Atg12-Atg16 (Czaja 2011). Protective mechanism of IL-22 in cerulein-induced pancreatitis involves the inhibition of autophagosome formation, which correlates with elevated levels of the anti-autophagic proteins Bcl-2 and Bcl-XL observed in the pancreatic tissues of IL-22 transgenic mice (Mareninova et al. 2009). The expression of Bcl-2 and Bcl-XL is likely influenced by IL-22, as the transcription of these proteins is regulated by STAT3, a signaling molecule known to be activated by IL-22 (Fortunato and Kroemer 2009; Gukovsky and Gukovskaya 2014). (Fig. 2)

Neutrophils and macrophages play a direct role in triggering intra-acinar cellular protease activation and acinar necrosis, which ultimately determine the severity of AP. Among these immune cells, neutrophils predominantly contribute to the entire process of SAP (Feng et al. 2012). IL-22 can indirectly inhibit the infiltration of inflammatory cells, especially neutrophils, and protect pancreatic acinar cells, whereas Reg3s play an irreplaceable and direct role in this process. Huan’s research demonstrated that the acinar cells with the highest IL-22 receptor expression levels exhibited enhanced IL-22 signaling in AP (Eyerich et al. 2009). This heightened signaling enables acinar cells to produce abundant Reg3s, which effectively counteract inflammatory cell infiltration and other pro-survival mediators to protect against tissue injury. Xue et al. additionally identified the importance of AhR activation in the induction of IL-22 (Mareninova et al. 2009), facilitating communication between immune cells and pancreatic acinar cells to provide protection against AP, including the alleviation of pancreatic acinar cell damage. In another study, histopathological improvements in the pancreas were also observed in the rIL-22 treated mice (Fu et al. 2016).

### IL-22 alleviate SAP-induced multiorgan injury

Acinar necrosis-induced inflammation at an early stage of acute pancreatitis plays a decisive role in the severity of the condition, potentially leading to SIRS and multiple organ dysfunction syndromes (MODS). Early phase mortality in patients with SAP is primarily attributed to multiple systemic organ failure (MSOF), with the lungs and intestines being the most vulnerable organs. Lung and intestinal injuries are major contributors to early mortality, resulting in a high mortality rate of 60% (Huan et al. 2016). IL-22 not only mitigates pancreatic acinar damage but also exhibits a protective effect against organ damage associated with SAP.

Inflammatory mediators including cytokines, chemokines, and reactive oxygen species play crucial roles in the development of AP-induced lung injury. These mediators contribute to the recruitment of macrophages and neutrophils, triggering a cascade of pathological changes in the pulmonary microcirculation, ultimately leading to the occurrence and exacerbation of lung injury associated with AP (Zhang et al. 2020). Compared to the PBS group, rIL-22 treatment stimulated the expression of Bcl-2, Bcl-xL, and IL-22R1 mRNA in lung tissues. Furthermore, administration of rIL-22 resulted in a significantly higher ratio of phosphorylated STAT3 (p-STAT3) to total STAT3 protein in the rIL-22 group than in the PBS group. Additionally, IL-22R1 expression increased following rIL-22 administration. These findings suggest that the remission effect of rIL-22 on associated lung injury in SAP mice is achieved by upregulating the expression of STAT3 signaling pathway-mediated anti-apoptotic genes such as Bcl-2 and Bcl-xL.

**Fig. 3:**
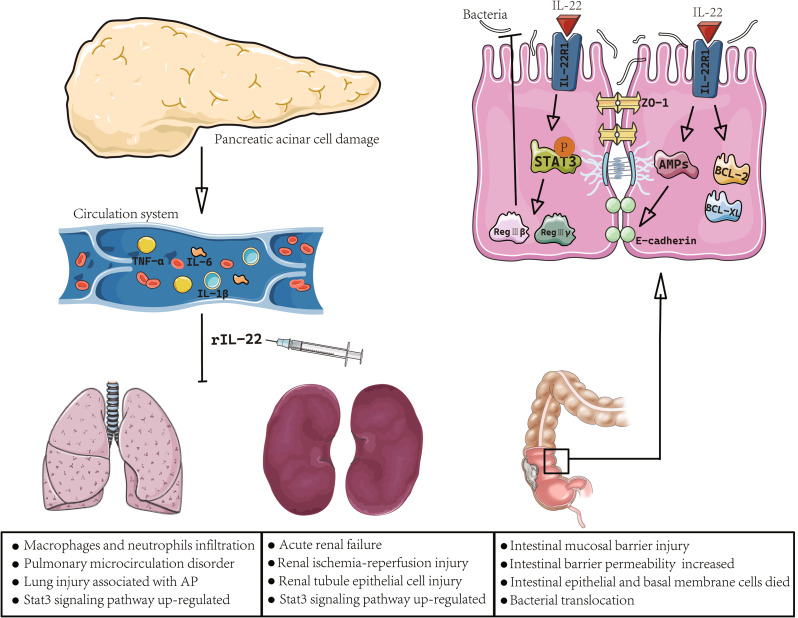
IL-22 alleviates SAP-induced multiorgan injury and intestinal barrier damage. The injury of pancreatic acinar cells leads to the release of numerous inflammatory mediators, including TNF-α, IL-6 and IL-1β, which enter the circulation system and reach the lung, kidney and intestine, initiating the recruitment and activation of immune cells in these organs, resulting in multiple organ damage, which can be alleviated after treatment with rIL-22. Besides, IL-22 induce STAT3 signaling to control bacterial growth in intestinal epithelial cells by enhancing the expression of RegIIIγ and RegIIIβ. IL-22 targets intestinal epithelial cells and induce of AMPs and Bcl-2 and Bcl-xL production. AMPs strengthen the mucus barrier by promoting tight junction proteins (ZO-1 and E-cadherin) production

Renal dysfunction is the most common external pancreatic organ injury in the early stages of SAP, followed by lung injury. Without intervention, this disease can rapidly progress to acute renal failure. Studies have shown that the mortality rate of patients with SAP complicated by kidney damage can reach more than 70%, and is one of the important causes of early death in patients with SAP (Kang et al. 2022; Samanta et al. 2018; Ge et al. 2020; Ma et al. 2011). Animal studies have shown that IL-22 can improve renal ischemia-reperfusion injury by protecting the epithelial cells of the renal tubules and reducing systemic inflammation (Kotenko et al. 2001). Injection of exogenous IL-22 has a protective effect on the renal function in L-arginine-induced SAP mice. IL-22 acts by binding to the specific receptor IL-22A1 on the mouse kidney, activating the downstream STAT3 signaling pathway, and upregulating the expression of protective factors such as Bcl-2 and BCL-XL to prevent apoptosis and necrosis of kidney tissues, thus protecting the physiological function of the kidney (Fig. 3).

### IL-22 protects SAP-induced intestinal barrier damage

In addition to parenchymal organ damage, impaired intestinal barrier integrity is detrimental to patients with SAP, resulting in the translocation of intestinal microbes and endotoxins. The excessive release of inflammatory cytokines during SAP is a significant contributing factor to intestinal barrier injury (Mathur et al. 2018). In SAP, intestinal injury occurs early and involves damage to the intestinal mucosal barrier and increased permeability. This increased permeability leads to extensive cell death in the intestinal epithelium and basement membrane, triggering a localized response and facilitating bacterial translocation outside the gastrointestinal tract. These events significantly contribute to MODS and systemic inflammatory response, ultimately damaging remote organs, but IL-22 can alleviate this phenomenon (Li et al. 2010, 2020; Herrera Gutiérrez et al. 2000). Several studies using murine models have demonstrated that IL-22-induced STAT3 signaling controls bacterial growth in intestinal epithelial cells by enhancing the expression of specific proteins, such as RegIIIγ (regenerating islet-derived protein 3) and RegIIIβ (Pasari et al. 2019). Besides, IL-22 has the capability to induce the production of antimicrobial proteins by various cells in the human body, including intestinal epithelial cells and keratinocytes. This process is a crucial component of the IL-22-mediated immune response in murine models (Dudakov et al. 2015; Garg and Singh 2019; Wang et al. 2020). A previous study revealed that the phosphorylation of STAT3 in intestinal epithelial cells regulates the immunological balance in the gut by enhancing IL-22-dependent mucosal wound healing [110]. Another study identified IL-22 as a crucial regulatory cytokine for maintaining gut epithelial integrity and mucosal immunity (Mareninova et al. 2009). IL-22 targets epithelial cells and exerts regulatory functions such as induction of the production of antimicrobial peptides (AMPs), strengthening the mucus barrier by promoting mucin production, enhancing epithelial regeneration via goblet cell restitution, and facilitating intestinal wound healing (Pickert et al. 2009). IL-22 expression is relatively low in the large intestines during normal physiological conditions, but it can be induced in response to inflammation (Mizoguchi et al. 2017). On the other hand, in the small intestine, IL-22 is constitutively expressed to maintain the integrity of the epithelial barrier (Dudakov et al. 2015). Studies by Endon et al. have demonstrated that IL-22 treatment effectively mitigates the increase in intestinal permeability and reduces the bacterial load in the gut following alcohol exposure (Protopsaltis et al. 2018).

IL-22 is a specific cytokine that primarily targets epithelial and stromal cells, connecting immune activation to colonic epithelial cells during intestinal inflammation (Ngo et al. 2018). In colonic tissues, the administration of rIL-22 enhances intestinal barrier function through various mechanisms. These include increased intestinal permeability, elevated expression of tight junction proteins (ZO-1 and E-cadherin), upregulation of antibacterial peptides (such as Reg3), and restoration of microbiota abundance (Jin et al. 2022a, b). Reg3, an antimicrobial peptide predominantly produced by intestinal epithelial cells, plays a vital role in bacterial elimination and barrier integrity. IL-22 induces the expression of Reg3 through the activation of the STAT3 signal in colon epithelial cells (Zheng et al. 2008). Additionally, exogenous rIL-22 treatment not only alleviates intestinal mucosal injury, including mucosal thickness, crypt depth, and villous height, but also upregulates the expression of Reg-IIIβ, Reg-IIIγ, Bcl-2, and Bcl-xL by activating the STAT3 signal as shown in a mouse model of L-arginine-induced SAP (Rendon et al. 2013). (Fig. 3)

## IL-22 acts interacts with other inflammatory mediators

CD4 + T cells, also known as T-helper cells, have previously been considered the primary suppliers of IL-22 in epithelial tissues. Among the various T-helper subsets, Th22 is an important subset and is enhanced by IL-21 or IL-26 (Geng et al. 2018). IL-22 serves as the main effector cytokine for the Th22 subset, and it is accompanied by the secretion of other effector cytokines such as IL-13, IL-26, and TNF-α (Gronke et al. 2019; Bai et al. 2021). Binding of IL-22 to its receptor, forming the IL-22-IL-22R1-IL-10R2 complex, activates Janus kinases (JAK) and Tyrosine kinases (TYK). JAKs phosphorylate STAT1, STAT3, and STAT5, as they are linked to IL-22R subunits (Lejeune et al. 2002). The stimulation of IL-22 in certain cells has been associated with the PI3K-Akt-mTOR and MAPK (JNK/SAPK, MEK-ERK-RSK, and p38 kinase) pathways (Lejeune et al. 2002; Matthews et al. 2011). (Fig. 1) IL-22 can also promote the production of IL-1, IL-8, and TNF-α (Bai et al. 2021; Gong et al. 2021). IL-6 and TNF-α up-regulate the aryl hydrocarbon receptor (AHR), which further binds to STAT-3 in the IL-22 promoter, promoting IL -22 transcription, whereas the hypoxia inducible factor-1 alpha (HIF-1a) inhibits IL-22 transcription (Blumenberg et al. 2017). RUNX3 and PGE2 have shown the potential to increase IL-22 expression (Mitra et al. 2012). An in vitro study has demonstrated that prostaglandin I analogs increase IL-22 levels and expand Th22 cells (Pan et al. 2022; Eyerich et al. 2009). Besides, a low concentration of IL-38 may suppress IL-22 production in cells, whereas a high quantity of IL-38 can boost Th22 cells to produce IL-22 (Fu et al. 2016; Niu et al. 2021). Studies in both mice and humans indicate that IL-22 orchestrates the inflammatory response by influencing the production of other proinflammatory cytokines, including IL-6, IL-8, and TNF-α (Yeste et al. 2014). Studies have suggested that the combined detection of serum IL-6 and hsa-miR-126-5p may help to predict the severity of AP early and that IL-6 acts as an independent risk factor in predicting the prognosis of AP patients (Li et al. 2020; Robb et al. 2018). Additionally, turtle (*Pelodiscus sinensis*) and Chinese pond turtle (*Chinemys reevesii*) peptides can enhance the IgA response and improve the intestinal barrier by activating the AHR/IL-22/STAT3/IL-6 axis (Garg and Singh 2019). This leads us to speculate on the ameliorative effect of the interaction between IL-6 and IL-22 in SAP. Further investigations are needed to clarify the interaction between IL-22 and other inflammatory factors in SAP.

## Conclusion

In this review, we discuss the effects of IL-22 on SAP and associated organ damage. Serum IL-22 level is positively correlated with the early stage of SAP patients, and significantly correlated with the degree of organ dysfunction and disease severity, which can be used as a potential early predictor. Mechanistically, on the one hand IL-22 alleviates pancreatic acinar damage by inhibiting autophagy and the infiltration of inflammatory cells, or promoting the production of reg3 to suppress inflammation. On the other hand, it also regulates innate immune defense and inflammation and promotes the production of antimicrobial peptides to slow the progression of multiple organ damage diseases associated with SAP. Timely detection of IL-22 is helpful to evaluate the pathogenesis and prognosis of SAP, and targeting IL-22 may provide a new therapeutic avenue for the treatment of SAP. However, the existing data are insufficient to identify the specific molecular mechanism. Therefore, further studies on the role of IL-22 in SAP are warranted.

### Electronic supplementary material

Below is the link to the electronic supplementary material.

Supplementary Material 1

## Data Availability

Yes.

## Abbreviations

AP: acute pancreatitis
AHR: aryl hydrocarbon receptor
Bcl-2: B-cell lymphoma-2
Bcl-XL: B-cell lymphoma-extra large
ERK: extracellular regulated protein kinases
HIF-1a: hypoxia inducible factor-1 alpha
IL-22: Interleukin-22
ILC3s: group 3 innate lymphoid cells
IL-22R: IL-22 receptor
IL-22BP: IL-22 binding protein
IFN-g: interferon-g
Jak1: janus kinase 1
JNK: c-Jun N-terminal kinase
MAPK: mitogen-activated protein kinase
MODS: multiple organ dysfunction syndrome
MSOF: multiple systemic organ failure
NK cells: natural killer cells
NKT cell: natural killer T cells
PGE2: prostaglandin E2
PBS: phosphate buffer saline
PI3K-Akt-mTOR: phosphatidylinositol 3- kinase- mechanistic target of rapamycin
rIL-22: recombinant IL-22
RAS: rat sarcoma
Reg3: regenerating islet-derived protein 3
RUNX3: RUNX family transcription factor 3 Gene
SAP: severe acute pancreatitis
SPAK: stress activated protein kinase
SIRS: systemic inflammatory response syndrome
Th cells: T help cells
Tyk2: tyrosine kinase 2
TNF-α: tumor necrosis factorα
ZO-1: zonula occludens-1
