# Supplementary figures and images for: The role of Interleukin-22 in severe acute pancreatitis
Source: Mol Med. 2024 May 15;30:60. doi: 10.1186/s10020-024-00826-7 (PMC11097471; doi:10.1186/s10020-024-00826-7)

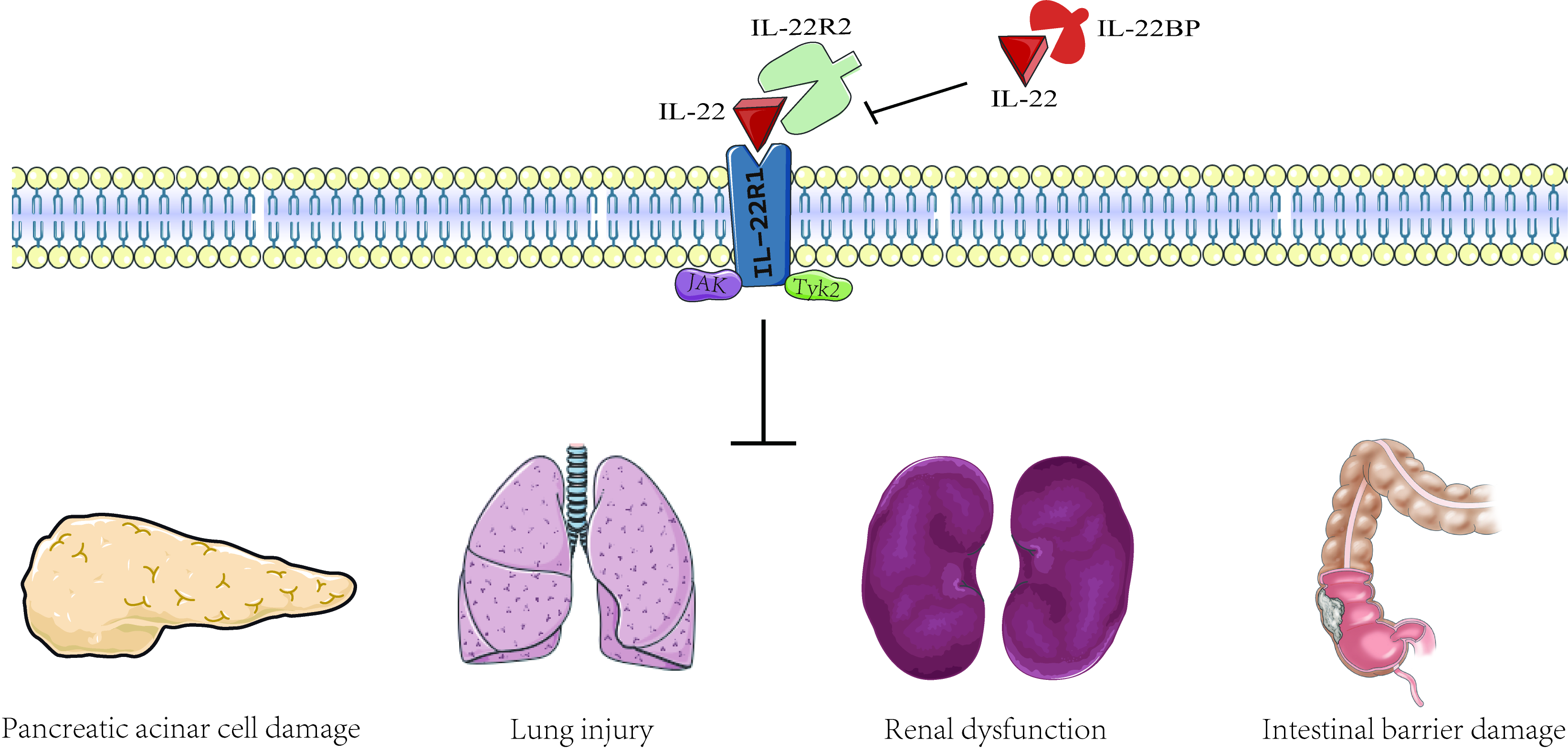

Supplement: Supplementary file 1 — Supplementary Material 1 [file 10020_2024_826_MOESM1_ESM.tif]
